# Early introduction of anakinra improves acute pericarditis and prevents tamponade in Staphylococcal sepsis

**DOI:** 10.1007/s11739-020-02627-2

**Published:** 2021-01-21

**Authors:** Ludovico Luca Sicignano, Maria Grazia Massaro, Marinica Savino, Donato Rigante, Laura Gerardino, Raffaele Manna

**Affiliations:** 1 Institute of Internal Medicine, Periodic Fever and Rare Diseases Research Centre, Fondazione Policlinico A. Gemelli IRCCS, Rome, Italy; 2 Università Cattolica Sacro Cuore, Rome, Italy; 3 Department of Cardiovascular and Thoracic Sciences, Fondazione Policlinico A. Gemelli IRCCS, Rome, Italy; 4 Department of Life Sciences and Public Health, Periodic Fever and Rare Diseases Research Centre, Fondazione Policlinico A. Gemelli IRCCS, Rome, Italy

## Abstract

The clinical response to anakinra observed by this patient concurrently treated with antibiotics indirectly confirms the potentially pathogenic role of IL-1 in maintaining the pericardial disease and shows how IL-1 blockade might allow avoiding the pericardiocentesis procedure. The report supports the hypothesis that anakinra is an effective and safe tool in the early treatment of acute pericarditis of presumed bacterial origin nonresponding to targeted antibiotic therapy.

Dear Editor,

Interleukin-1 (IL-1) is known to be a pleiotropic cytokine with a master role in the genesis of pericarditis [1], and the recombinant IL-1 receptor antagonist anakinra is currently considered a standard treatment for recurrent or refractory idiopathic pericarditis, especially for the corticosteroid-dependent cases [2]. The effects of anakinra in the initial phase of acute pericarditis have not been studied.

Herein, we present the case of an 87-year-old man, who was independent in his activities of daily living, but suffering from systemic arterial hypertension and chronic renal failure (creatinine clearance: 45.25 ml/min). This patient had a long-lasting severe eczematous dermatitis sparing only the face and came to our Emergency Department due to high-spiking fevers and shivering. Lab works showed increased inflammatory markers and neutrophil leukocytosis. Given the positivity of his blood cultures for a methicillin-sensitive *Staphylococcus aureus*, also found on his nose and skin swabs, a targeted antibiotic treatment with oxacillin was prescribed. He also complained of chest pain suggestive of acute pericarditis, and echocardiography revealed a moderately severe circumferential pericardial effusion (24 mm in the anterior site; 15 mm adjacent to the right atrium, and 21 mm in the postero-lateral site) with signs of systolic collapse of the right atrium and mild diastolic collapse of the free wall of the right ventricle (Fig. 1: panels a–c). Electrocardiogram showed sinus rhythm, ventricular extrasystoles, left anterior hemi-block and left ventricular hypertrophy with ST-T abnormalities, but no other specific signs of pericardial diseases. Anti-inflammatory therapy with ibuprofen (200 mg t.i.d.) and colchicine (1.5 mg/day) was started. Screening for latent tuberculosis was negative. After 5 days, the large and partially organized pericardial effusion increased on echocardiography, and chest pain worsened. Furthermore, anti-toxic shock syndrome toxin-1 immunoglobulins E were found in patient’s blood (6.83 U/ml), confirming the Staphylococcal role on his disease. In consideration of this scenario, before considering pericardiocentesis, anakinra was started at the dosage of 100 mg/day subcutaneously. One day later, all painful symptoms subsided. At the following echocardiogram, performed after two anakinra doses, pericardial effusion was substantially reduced (7 mm in the anterior site; 11 mm in the right atrial area, and 15 mm in the postero-lateral site); no signs of collapse of the right atrium and right ventricle were documented (Fig. 1: panels d–f). Evacuative pericardiocentesis was, therefore, avoided. In addition, leukocytosis improved and inflammatory markers turned to normal in a few days. The patient was discharged after 14 days of antibiotic therapy and 5 days of anakinra (which was then stopped), and is now in overall good health, still receiving colchicine (1 mg/day). Anakinra neither induced any immune suppression, nor side-effects and did not prevent the complete recovery from the *Staphylococcus aureus* acute infection.

**Fig. 1:**
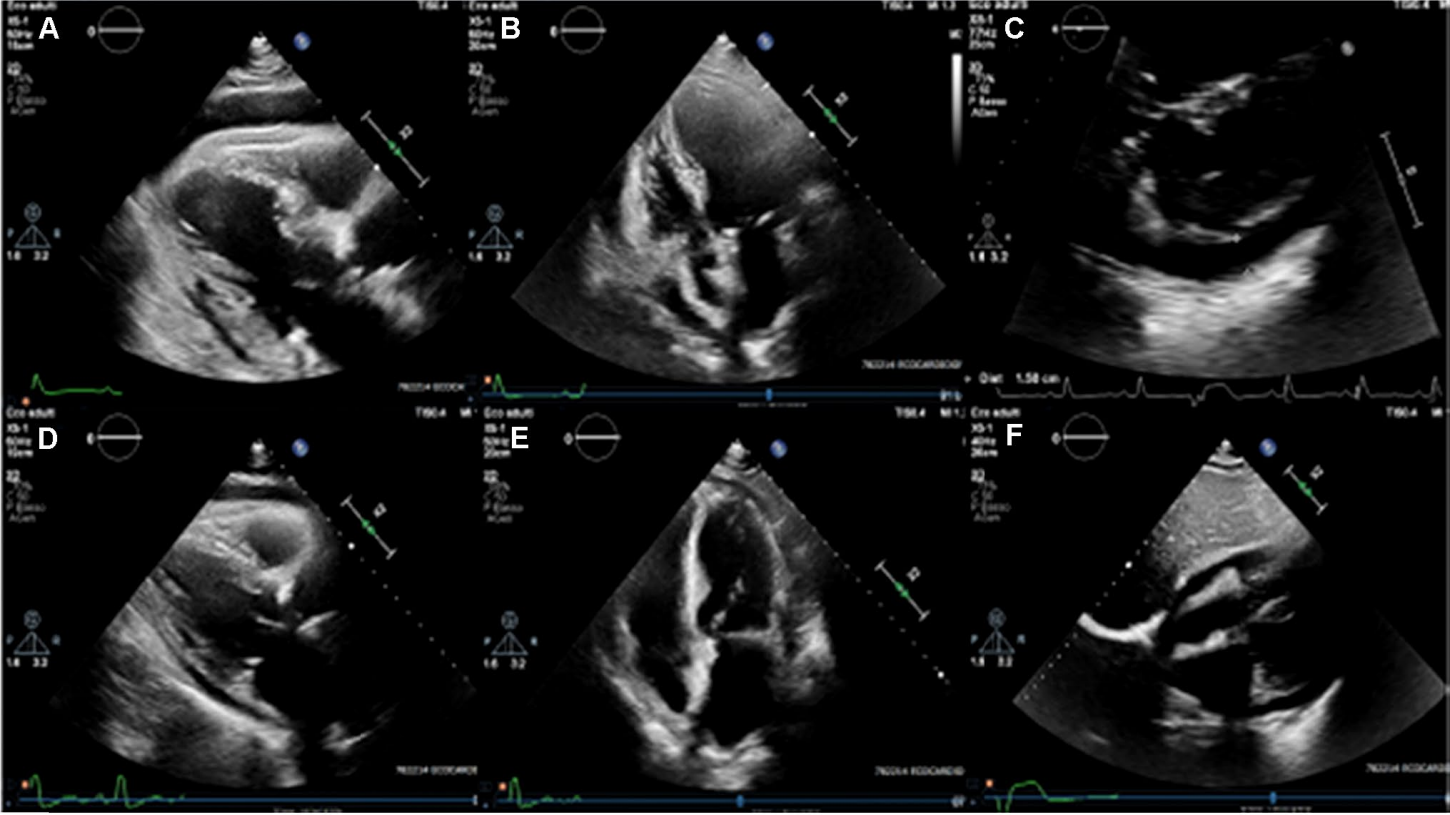
Echocardiographic assessment of pericardial effusion. **a**–**c** Diastolic frames of parasternal long axis, 4-chamber and subcostal views, respectively, acquired at our first evaluation. A moderately severe circumferential pericardial effusion is evident with signs of initial collapse of the right atrium. **d**–**f** The same views acquired after treatment with anakinra was started: pericardial effusion appears substantially reduced with no more signs of right atrium collapse

Complications arising from life-threatening invasive infections caused by *Staphylococcus aureus* are a major clinical problem owing to their high incidence and widespread emergence of antibiotic-resistant bacterial strains. The identification of specific immune impairments in humans that predispose to *Staphylococcus aureus* infections, for instance a breakage in the epidermal barrier for subjects with chronic inflammatory dermatoses, has provided new insights into the key immune responses that are involved in invasive infections. Pericarditis caused by *Staphylococcus aureus* is a rare event [3]. As with other types of bacterial pericarditis, antibacterial therapy should be started immediately, but chest pain may often persist for days despite an adequate treatment. Concurrently, a timely pericardial drainage maybe essential in a subset of patients displaying severe pericarditis with significant effusion giving rise to hemodynamic compromise [4]. The use of non-steroidal anti-inflammatory drugs and colchicine is currently the first-choice therapy in acute pericarditis combined with targeted antibacterial therapy if a bacterial origin is presumed [5]. Early use of colchicine has also resulted effective in the resolution of recurrent polyserositis [6]. Conversely, IL-1 inhibitors are frequently called on idiopathic recurrent acute pericarditis [7], and indeed recent trials have shown that anakinra is effective for the treatment of idiopathic recurrent pericarditis and that the IL-1 blocking agent canakinumab is even effective in reducing myocardial infarction rates when given to high-risk patients [8]. Therefore, anti-IL-1 agents have become the first-class tools in the treatment of several autoinflammatory conditions [9, 10]. In addition, IL-1 antagonists have determined a paramount clinical response even on the neurologic features of the most complex autoinflammatory disorders [11]. Nevertheless, little is known about early use of anakinra in acute pericarditis, especially in patients proved to have a bacterial origin of their pericardial disease. Very recently, Wohlford et al. reported 5 patients with acute pericarditis meeting the European Society of Cardiology criteria who had moderate-to-severe chest pain within 24 h of presentation and within 6 h of the clinical diagnosis being made, who were early treated with subcutaneous anakinra (100 mg), whereas non-steroidal anti-inflammatory drugs and colchicine were suspended for 24 h. Four of the 5 patients enrolled were men, 3 were Caucasian and their median age was 38 years. The authors showed that the first dose of anakinra caused both reduction of cytokine production and reduction of neutrophil leucocytosis, but above all, the resolution of painful pericardial symptoms soon after 6 h: the reduction in chest pain intensity paralleled the significant reduction in serum IL-6 levels after 24 h from anakinra [12]. This proof-of-concept trial did not include any patients with pericarditis of bacterial origin and did not consider any possible contribution of anakinra on the resorption of pericardial effusions or on a future reoccurrence of pericarditis, but disclosed the importance of IL-1 in the acute pericarditis syndrome, opening the door to a broader exploration of IL-1 blocking agents.

It is established that *Staphylococcus aureus* can induce both biosynthesis and release of IL-1, which has a pivotal role in the serosal inflammation and T cell polarization. Ikejima et al. revealed that certain strains of *Staphylococcus aureus* associated with toxic shock syndrome induce human blood monocytes to secrete IL-1 in vitro, and IL-1 was detected both by its ability to cause fever in rabbits using the leukocytic pyrogen assay and by its mitogenic activity towards thymocytes in the so-called lymphocyte-activating factor assay. Moreover, the stimulation of monocyte IL-1 production was easily quantified, providing a simple method of assaying the toxic shock syndrome toxin. The authors argued that both sudden fever and other components of the acute phase response may be attributed to a massive release of IL-1 in the case of an infection caused by toxic shock syndrome toxin-producing *Staphylococcus aureus* [13].

IL-1 is also one of several proinflammatory cytokines produced during most infections and mostly during sepsis, and the systemic inflammatory response syndrome that serves to initiate the host response and integrate nonspecific immunity may be beneficial to the host, with promotion of neutrophil recruitment and anti-microbial peptide release: however, if produced for extended periods of time or in excessive quantity, IL-1 might contribute to morbidity and mortality. Indeed, during sepsis, dysregulation of the finely tuned balance between IL-1 production and its inhibition may result in severe inflammation, tissue damage and organ dysfunction. In fact, uncontrolled IL-1 production has been directly linked to increased release of prostaglandins (PGE_2_ and PGI_2_), platelet-activating factor and nitric oxide combined with tissue neutrophil infiltration and subsequent increased vascular smooth muscle relaxation, vascular leak, development of hypotension, shock and multi-organ failure [14]. Not by chance, recent research interests have been focusing on IL-1 inhibition to improve outcome in sepsis and septic shock. The inhibition of IL-1 actions should theoretically prevent or at least attenuate many of these responses and probably lead to decrease morbidity and mortality.

Among biological treatments, the IL-1 receptor antagonist anakinra has demonstrated a good safety profile, and the general infection rate associated with its use has resulted not significantly increased in patients with rheumatoid arthritis on anakinra. Nevertheless, an increased risk was observed for higher doses of anakinra (> 100 mg) in patients with comorbidity factors. Thus, anakinra administration in daily practice should require careful monitoring, especially in patients with comorbidities and concomitant treatments, such as glucocorticoids [15].

The clinical response to anakinra observed by our patient concurrently treated with antibiotics indirectly confirms the potentially pathogenic role of IL-1 in maintaining the pericardial disease and shows how IL-1 blockade might allow avoiding the pericardiocentesis procedure. Our report supports the hypothesis that anakinra is an effective and safe tool in the early treatment of acute pericarditis of presumed bacterial origin nonresponding to targeted antibiotic therapy. On the other hand, our positive experience should not encourage overlooking caution when administering anakinra to patients with a history of any infections or with underlying conditions which may predispose to infections. Interesting will be to investigate whether early IL-1 blockade could play a role in the risk of relapses after a first episode of acute pericarditis.
